# Rocket Science: The Effect of Spaceflight on Germination Physiology, Ageing, and Transcriptome of *Eruca sativa* Seeds

**DOI:** 10.3390/life10040049

**Published:** 2020-04-24

**Authors:** Jake O. Chandler, Fabian B. Haas, Safina Khan, Laura Bowden, Michael Ignatz, Eugenia M. A. Enfissi, Frances Gawthrop, Alistair Griffiths, Paul D. Fraser, Stefan A. Rensing, Gerhard Leubner-Metzger

**Affiliations:** 1 Department of Biological Sciences, Royal Holloway University of London, Egham TW20 0EX, UK; jake.chandler@rhul.ac.uk (J.O.C.); k_safina@hotmail.com (S.K.); ignatz.michael@gmail.com (M.I.); Genny.Enfissi@rhul.ac.uk (E.M.A.E.); P.Fraser@rhul.ac.uk (P.D.F.); 2 Plant Cell Biology, Faculty of Biology, University of Marburg, 35043 Marburg, Germany; fabian.haas@biologie.uni-marburg.de (F.B.H.); stefan.rensing@biologie.uni-marburg.de (S.A.R.); 3 Official Seed Testing Station for Scotland, SASA, Edinburgh EH12 9FJ, UK; Laura.Bowden@sasa.gov.scot; 4 Tozer Seeds Ltd, Cobham, Surrey KT11 3EH, UK; Frances.Gawthrop@tozerseeds.com; 5 Science Department, Royal Horticultural Society, Woking, Surrey GU23 6QB, UK; alistairgriffiths@rhs.org.uk; 6 Laboratory of Growth Regulators, Centre of the Region Haná for Biotechnological and Agricultural Research, Institute of Experimental Botany, Academy of Sciences of the Czech Republic, Palaćky University, 78371 Olomouc, Czech Republic

**Keywords:** climate change, food security, low Earth orbit, salad rocket (*Eruca sativa*), seed germination, spaceflight transcriptomes, seed ageing, seeds in space, seed storage, seed vigor

## Abstract

In the ‘Rocket Science’ project, storage of *Eruca sativa* (salad rocket) seeds for six months on board the International Space Station resulted in delayed seedling establishment. Here we investigated the physiological and molecular mechanisms underpinning the spaceflight effects on dry seeds. We found that ‘Space’ seed germination vigor was reduced, and ageing sensitivity increased, but the spaceflight did not compromise seed viability and the development of normal seedlings. Comparative analysis of the transcriptomes (using RNAseq) in dry seeds and upon controlled artificial ageing treatment (CAAT) revealed differentially expressed genes (DEGs) associated with spaceflight and ageing. DEG categories enriched by spaceflight and CAAT included transcription and translation with reduced transcript abundances for 40S and 60S ribosomal subunit genes. Among the ‘spaceflight-up’ DEGs were heat shock proteins (HSPs), DNAJ-related chaperones, a heat shock factor (*HSFA7a-like*), and components of several DNA repair pathways (e.g., *ATM*, *DNA ligase 1*). The ‘response to radiation’ category was especially enriched in ‘spaceflight-up’ DEGs including HSPs, catalases, and the transcription factor *HY5*. The major finding from the physiological and transcriptome analysis is that spaceflight causes vigor loss and partial ageing during air-dry seed storage, for which space environmental factors and consequences for seed storage during spaceflights are discussed.

## 1. Introduction

Plant science research during spaceflight, including on orbiting space platforms such as the International Space Station (ISS), has revealed novel mechanisms and the potential of ‘dry’ seeds, growing seedlings, and flowering plants to respond to environmental change and extreme conditions outside their evolutionary history [1,2,3,4,5]. These findings are relevant for sustainable agriculture, climate change, food security, and seed storage on the Earth’s surface, as well as for growing fresh food during long-distance space travel and for agriculture on Mars and other planets [1,6,7,8]. Laboratories inside spaceships in low Earth orbit (LEO) have enabled investigations of the effects of long-term microgravity and helped, for example, to unravel mechanisms underpinning plant tropisms that would otherwise be masked by the effects of gravitropism [2,9]. Molecular analysis of growing seedlings and adult plants in microgravity by epigenomics [10], transcriptomics, and proteomics [8,9,11] identified known and novel genes specific for the response to the space environment. This research has potential to be applied to growing fresh food derived from plants for nutrition and human survival in space during long-distance space travel [5,7,12,13,14,15,16,17]. These works demonstrated that successful ‘seed-to-seed’ plant cultivation and propagation is possible but also that space-produced seeds may differ in reserve composition and properties. Long-term exposure to microgravity inside spaceships also led to the important discovery that it is associated with accelerated ageing of humans and plants [4].

In many organisms, including most plants, the passage through arrested life stages is associated with desiccation tolerance, the ability to survive extreme water losses by expressing protection mechanisms for the low-hydrated tissues and macromolecules in the ‘air-dry’ state [18,19,20,21,22]. Plant species with desiccation tolerance of vegetative tissues (usually 80–90% water content) are rare, yet it is common in plant seeds, which in their dry state have 5–10% water content. Low ambient humidity and low temperature are the recommended long-term dry storage conditions for desiccation-tolerant plant seeds in ex situ seed banks and seed company warehouses. Crop seed quality is compromised by ageing during seed storage, which initially manifests as reduced seed vigor (germination performance) and subsequently as reduced seed viability (seed death). Seed ageing during storage is caused by oxidation of macromolecules (proteins, lipids, nucleic acids, etc.) leading to progressive deterioration [20,23,24]. The ability to repair accumulated oxidative damage during seed imbibition (antioxidant systems, DNA ligases, etc.) and the biochemical, biomechanical, and micromorphological properties of the protecting seed and fruit coat layers are important determinants of seed quality [18,21,25,26,27]. Viability loss is a late response during dry storage preceded by vigor loss due to ageing processes. The use of controlled artificial ageing assays provided insight into the molecular processes underpinning the loss of seed vigor and viability.

A number of studies have investigated seed viability during long-term exposure to the harsh space environment without and with shielding against space radiation [1,5,28,29,30,31]. *Arabidopsis thaliana* (Arabidopsis) and tobacco seed lots exposed for >>1 year to the outside space of the ISS (LEO ~450 km altitude, temperatures fluctuating between −25 and 50 °C, vacuum 10^−4^ to 10^−7^ Pa, space radiation absorbed dose 350–400 mGy) showed delayed germination (reduced germination speed), but with ~90% viability when shielded from short-wavelength ultraviolet (UV) radiation (>1 GJ m^−2^ UV_110–400 nm_) [30,31]. When exposed unshielded, the short-wavelength UV radiation caused a severe reduction in seed lot viability. Ground simulation (room temperature, normal pressure (10^5^ Pa), 1−3 µGy day^−1^) confirmed that ~1 GJ m^−2^ UV_254 nm_ is indeed a lethal dose for Arabidopsis and tobacco seeds but not for field bindweed seeds, which have a hard seed coat with phenolic pigments potentially providing the protective shielding against the UV radiation. The finding that shielding against short-wavelength UV radiation retains viability was also found to be true for dry seeds of other plant species and for desiccation-tolerant bacterial and fungal spores; also, there seem to be species-specific differences in the self-shielding properties by phenolic pigments in seed and spore coats (see review and original references in [1]). The delay observed in Arabidopsis seed germination depended upon spacecraft position, probably owing to differences in radiation, with positions that were more shielded resulting in effects of lesser intensity [32]. Long-term exposure of cereal grains (rice, wheat, barley, corn) to the outer space environment with different degrees of shielding confirmed in principle their viability and capability to produce healthy seedlings. However, specific molecular damage by the space environment also occurred in the cereals and resulted in delayed and reduced germination [33], reduced photosynthetic performance of seedlings [34], and spaceflight-induced mutations affecting stress tolerance genes [35]. The best shielding for crop seed transport during long-distance space travel therefore seems to be seed storage inside spaceships. 

On 2 September 2015, 2 kg of rocket seeds (*Eruca sativa*) were sent to LEO aboard Soyuz TMA-18M to spend six months on board the ISS, returning to Earth on 2 March 2016, as part of the ‘Rocket Science’ project [36]. This project was initiated by the Royal Horticultural Society (RHS) in collaboration with the UK Space Agency and supported by the European Space Agency (ESA) with the British astronaut Tim Peake. It involved over 5000 schools in the UK who undertook set experiments to determine if the spaceflight affected germination and seedling establishment. The results of the experiments undertaken by schools suggested that spaceflight may cause a minor delay in seedling emergence and growth [36]. The seed samples also represented a unique opportunity to investigate the subtle effects of seed storage alongside human spaceflight (i.e., seed stored inside pressurized cabins) on germination physiology and the seed transcriptome. Here we investigate the effects of this spaceflight, which included six months on board the ISS, on the germination physiology, dry seed transcriptome, and response to controlled artificial ageing treatment (CAAT) of rocket seeds.

## 2. Materials and Methods 

### 2.1. Seed Material, Space Travel and Packaging

**Figure 1:**
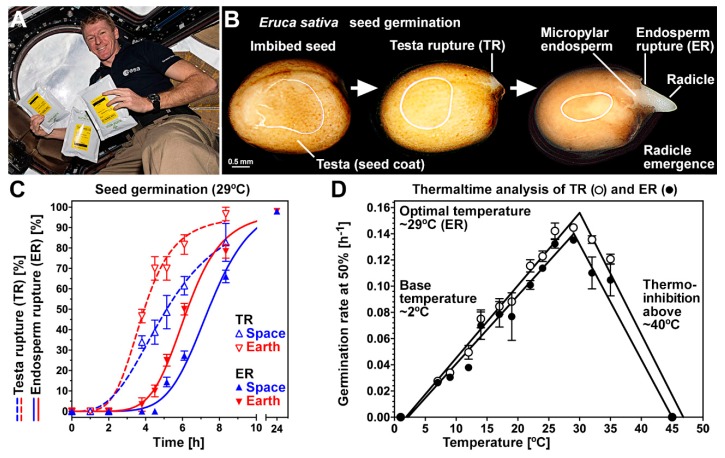
Effect of spaceflight on germination of *Eruca sativa* (rocket) seeds. (**A**) The Space seed batch was stored on the ISS for six months, sealed at low humidity in foil bags; Image: ESA/NASA. (**B**) Visible stages of rocket seed germination. (**C**) Time course analysis of testa rupture and endosperm rupture of Earth seeds compared to Space seeds. (**D**) Thermal time modeling of Earth-WS (warehouse stock) seed germination over a range of temperatures. Note that germination rates were calculated as the inverse of the time required to reach 50% germination of the population at the respective temperature. Mean of three replicates of 20 seeds per plate. Error bars indicate ± standard error.

Seeds of salad rocket, *Eruca sativa* Mill. (TZ 5007 seed lot 09835147, Tozer Seeds Ltd. Pyports, Downside Bridge Road, Cobham, Surrey, KT11 3EH, UK) were used in the Rocket Science project [36]. Company warehouse storage of this seed lot was at 14 °C with a relative humidity (RH) of 25% in foil bags. Three seed batches originating from this seed lot were used in our experimental work (depicted as flow chart in Figure S1): (1) The seed batch ‘Earth-WS (Warehouse Stock)’ was directly taken from this seed lot stored in the described warehouse conditions (14 °C, 25% RH, Figure S1). The Earth-WS seed batch therefore provides a ground control which remained in the warehouse storage and therefore directly represents the original seed lot (no space journey, no packaging process). (2) The seed batch ‘Earth’ also provides a ground control, but in addition to the several-month warehouse storage (~22 °C, 25% RH, Figure S1) it also underwent the packaging process in March 2016. In this packaging process, individual foil packets (red) were filled with 100 seeds each (Seed Packers Ltd., Bury St Edmunds, Suffolk, IP32 6NL, UK). (3) The seed batch ‘Space’ underwent the space journey (Figure S1) and subsequently the same packaging process in March 2016 to provide individual foil packets (blue). During the entire space journey, seed were transported and stored in sealed foil bags with a humidity of 25% inside a jiffy envelope (Figure 1A). While Space seeds underwent the space journey (plus packaging), Earth-WS (no packaging) and Earth (plus packaging) remained in warehouse storage. Packaging of the Space and Earth seeds was conducted under the same conditions and with the same foil bags (red and blue) in the second half of March 2016.

For the space journey (Figure S1), Space seed were transported in the sealed foil bags (Figure 1A) by airplane from London (UK) to Baikonur (Kazakhstan) via Noordwijk (Netherlands) and Moscow (Russia) in August 2015. The transport was at room temperature (18–25 °C), but as no temperature datalogger was used, we cannot exclude occasional fluctuations beyond this temperature range. Space seeds were launched aboard Soyuz 44S (air temperature on board 18–25 °C) to the ISS on 2nd September 2015. Space seeds were stored for six months inside the ISS (with an average temperature of 22 to 23 °C and occasional fluctuations between 18 and 32 °C), and the location within the space station changed; the seeds were stored most of the time in the Columbus laboratory and occasionally were removed for photographs (still sealed within foil bags, Figure 1A). Space seeds boarded the Soyuz capsule on 2nd March 2016 for re-entry to Earth, landing on the Kazakh Steppe. Seeds were transported to London (UK) by airplane via Moscow (Russia), Stavanger (Norway), and Houston (USA) and underwent the packaging process in the UK starting 14 March 2016. The sealed foil bag during the space journey was to maintain humidity (low), air composition, and pressure throughout. Moisture content was determined by drying 100 mg of seed in glass vials using a drying oven for 17 h at 103 °C and reweighing. Dry storage of the seeds in red and blue foil bags in the Seed Science Laboratory at Royal Holloway University of London was at 22 °C in a sealed plastic container above silica gel (generating <15% RH). Conductivity assays were conducted in May 2016, CAAT assays took place January–April 2017, and RNA extraction for the transcriptome analysis was in May 2017. Germination assays were conducted throughout the dry storage period and consistently demonstrated that the Space seeds germinated slower (Figure 1).

### 2.2. Controlled Artificial Ageing Treatment (CAAT)

Seeds were transferred to 2 mL Eppendorf tubes and exposed to 70% RH (in the headspace of a sealed container above 25 g/100 mL LiCl) for three days at 22 °C and then exposed to 35 °C for the specified number of days (length of CAAT). Seeds were then dried above silica beads at 35 °C for 2 h and subsequently stored in sealed vials at room temperature until germination assays were carried out. CAAT is a commonly used method in seed science and industry to compare the ageing sensitivity of seeds during dry storage. To achieve this in an experimentally reasonable time, the combination of increased temperature (usually to 35–45 °C) and RH (usually 70–90% to generate an elevated seed moisture content) is required [19,22,37].

### 2.3. Germination Assays

Seeds were imbibed on two layers of filter paper in 6 cm Petri dishes, using two layers of ⌀ 5 cm filter paper disks and 3 mL distilled water, with 20 seeds per replicate. Plates were incubated in continuous white light (~100 µmol sec^−1^ m^−2^) in a Panasonic MLR-352 Environmental Test Chamber (ETC, Panasonic, Bracknell, UK) at the temperatures indicated. In order to conduct the thermal time modeling (Figure 1D) seeds were imbibed on a thermogradient plate with a temperature range between 0 and 45 °C under constant white light (Grant Gradient Plate GRD-1, Grant Instruments (Cambridge) Ltd., UK). Seed germination was scored over time as either testa rupture, endosperm rupture, or visible radicle protrusion (completion of germination), as indicated. 

### 2.4. Conductivity Assay

Four replicates of 100 seeds of both Space and Earth seeds were weighed and added to 100 mL beakers containing 50 mL distilled water and stirred to ensure that all seeds were submerged. Beakers were covered and placed at 20 °C. Electrical conductivity (EC) [38] of solutions was measured using a Jenway 4510 conductivity meter (Cole-Parmer, Stone, UK), and conductivity was calculated according to the following equation: EC = (conductivity of seed soak solution (µS cm^−1^) – conductivity of control solution (µS cm^−1^)) / weight of replicate (g).

### 2.5. RNA Extraction and Sequencing

Total RNA was extracted from dry or CAAT seeds for each of Earth-WS, Earth, and Space seed batches, three replicates per treatment, as outlined previously [39]. Its quality was verified using an Agilent Bioanalyzer 2100. RNA libraries of the 18 samples were sequenced using a HiSeq V4 sequencer (Illumina) generating paired-end 125 bp reads (Vienna BioCenter Core Facilities GmbH, Austria).

### 2.6. Sequencing Quality Control, de Novo Assembly and Differentially Expressed Gene (DEG) Detection

Sequencing quality was checked with FastQC version 0.11.05 [40]. Read quality trimming and adapter removal was performed using Trimmomatic 0.36 [41] with the following parameters: ILLUMINACLIP:TruSeq3-PE-2.fa:2:30:10, SLIDINGWINDOW:4:15, and MINLEN:50. Organellar reads were filtered using GSNAP version 2018-05-0 [42] to map reads against the mitochondrial genome of *Eruca sativa* (NCBI KF442616.1), chloroplast (NCBI KU050690.1, KT581449.1, and KY88366.3) and ribosomal (NCBI KF442616.1, KT626568.1, HM041514.1, KX358569.1, LC090005.1, X63524.1, LN589682.1, AB511961.1, KX282137.1, and JX444504.1) sequences of closely related organisms. To remove read contamination, DIAMOND [43] version 0.9.22 was used to align reads against the NCBI nr database (BLASTx, downloaded 18 October 2018). Results were analyzed using MEGAN [44] version 6.12.5. All reads clustered to Opisthokonta were removed. This was done for forward and reverse reads, and respective pairs were also removed. Reads contributing to an aberrant 35% GC peak (suspected contaminating reads not from *E. sativa*) in forward reads were removed by filtering forward reads with 32% to 39% GC content and their respective pairs. Trimmed and filtered read pairs were used to run a de novo transcriptome assembly using Trinity [45] version 2.2.6. The option ‘--min_contig_length 300’ was used in addition to the default settings. BUSCO version 3.0.1 was used to determine completeness of the transcriptome with 84.2% completeness for Viridiplantae observed [46]. Assembled transcripts were annotated using BLASTx with the NCBI nr database running on DeCypher 9.0.0.25, tera-BLASTx 9.0.0 [47]. Gene ontology (GO) terms for the transcripts were assigned using Blast2GO version 2.5 (https://www.blast2go.com) [48] with ca. 10,000 transcripts per batch.

Read pairs, used for de novo transcriptome assembly, were mapped against the longest assembled transcripts using GSNAP with the additional parameters -N 1 and --npaths 7. Subread feature Counts version 1.6.2. [49] was used to count the reads mapped on the longest assembled transcripts with the parameters: -p -B -t exon -g Parent. The full transcript length was used as an exon in an artificial GFF3 annotation file. NOISeqBIO [50] was used with the parameters norm = “rpkm”, lc =1, r = 20, a0per = 0.9, and cpm = 15 to determine DEGs between samples in a pairwise manner. The Space dry seed sample replicate 2 was determined to be an outlier and was not used in the analysis. For downstream analyses, only transcripts with top BLAST hits in Viridiplantae were used. Taxonomic IDs were filtered in R 3.4.2 using the package taxize [51]. GO term enrichment was analyzed with the topGO Bioconductor package version 2.34.0 [52] using the classic method and the Fisher test. The top GO terms were clustered by enrichment score (1/log_10_(p)) using one minus Pearson’s correlation coefficient and visualized in Morpheus [53]. 

### 2.7. Carotenoid Tocopherol and Chlorophyll Determinations

The carotenoid and chlorophyll pigments and antioxidant tocopherols were extracted and quantified as described in Drapal et al. [54]. Expanding leaf material was freeze-dried using a FreeZone LABCONCO, VWR. The dried leaf material was ground into a fine homogeneous powder using a Qiagen, TissueLyser LT with 1 min shaking at an oscillation frequency of 50 Hz. Extraction was performed from dried, powdered tissue (10 mg) placed in a microcentrifuge tube (2 mL); 100 mM Tris-HCl pH 8.0 (400 µL) and methanol (400 µL) were added, and the mixture was shaken using a vortex. The suspension was rotatory inverted for 1 h at 4 °C in the dark. Chloroform (800 µL) was added to the suspension before mixing with the vortex (three times, 5 s). This extraction method is adapted from the classic Bligh and Dyer method [55]. The mixture was then centrifuged at maximum speed (10,000 *g*) for 3 min in a microcentrifuge to facilitate the formation of phase separation. The lower organic layer (hypophase) containing the isoprenoids was recovered and transferred to a fresh 1.5 mL tube, and the aqueous phase was re-extracted with an equal volume of chloroform. The combined organic phases were dried under a stream of N_2_ gas. UPLC-PDA was used to separate, identify, and quantify the pigments. The dried samples were re-dissolved in ethyl acetate (50 µL) and centrifuged at maximum speed for 5 min to remove potential precipitate from the sample. The clean extract (30 µL) was transferred to a ULPC glass insert previously mounted in a glass vial and capped, from which 3 µL was injected onto the UPLC. Chromatographic separation was carried out in a reverse phase mode using a C18 column (50 × 2.1 mm i.d., 1.8 µm) and a linear gradient of mobile phases A (methanol/water (50:50, v/v)) and B (acetonitrile (ACN)/ethyl acetate (75:25, v/v)). The column operated at 30 °C with a flowrate of 0.6 mL/min; automated sample temperature was kept at 8 °C. Detection was performed using a UV/Vis scan ranging from 200 to 600 nm. Comparative absorption spectrum and retention times with authentic standards were used to identify individual carotenoids, chlorophylls, and tocopherols [56]. Quantification was achieved from dose–response curves using authentic standards.

## 3. Results

### 3.1. Germination of Rocket Seeds Following Spaceflight

Seeds of *Eruca sativa* (rocket) from the Brassicaceae plant family were used in the experiments. The ‘Earth-WS (Warehouse Stock)’ ground control seed batch that had neither been packaged nor flown in space was used to establish the temperature dependence of rocket seed germination (Figure 1). Following seed imbibition, the first visible step in rocket seed germination is the rupture of the outermost nonliving seed coat layer, the testa (Figure 1B). Testa rupture (TR) exposes the living endosperm layer underneath, which covers the radicle (embryonic root) at the seed’s micropylar end. Following TR is endosperm rupture (ER), which marks the completion of germination and allows radicle emergence (Figure 1B) and subsequent embryo and seedling growth. Rocket seeds therefore exhibit a two-step process of visible events during their seed germination, as is known from other Brassicaceae species including Arabidopsis and garden cress, as well as from tobacco [18,27,57]. By imbibing rocket seeds at a range of temperatures, the optimum germination temperature was found to be ~29 °C (Figure 1D). Germination was found to be fastest at this temperature, leading to maximized germination rate (GR_50%_), which is the inverse of the average time to 50% germination (t_50%_) of the seed population. Thermal time modeling revealed a maximum germination percentage of 95–100% over a wide range of temperatures (7 to 35 °C), indicating that seeds were nondormant. For the Rocket Science project [36], rocket seeds from the Space batch sealed in air- and humidity-tight seed storage foil bags were launched into space for a six-month stay on board the ISS (Figure 1A). Upon return, these space-flown seeds underwent the packaging process, which provided packets each containing ~100 ‘Space’ seeds; ground control seed packaging provided ‘Earth’ seed packets. A flow chart depicting the relationship between the three seed batches ‘Earth-WS’, ‘Earth’, and ‘Space’, as well as their storage conditions is presented in Figure S1.

The three seed batches, Space (blue packets), Earth (red packets) [36], and Earth-WS (14 °C and no packaging process), were comparatively analyzed. Air-dry seeds in the packets equilibrated to a significantly different moisture content of 8.0% ± 1.0% for Space seeds and 6.3% ± 0.1% for Earth seeds; Earth-WS and Earth seeds had equal values (Figure S2). Figure 1C shows a comparison of the germination characteristics of Space and Earth seeds at 29 °C. The times for TR and ER were significantly delayed by 2.0 and 1.6 h, respectively, in Space seeds as compared to Earth seeds (Figure 1C). Nonetheless, both Space and Earth seeds had a final germination percentage of ~100% without any abnormal seedlings, indicating that space travel did not reduce seed viability. Space and Earth rocket seedlings developed normally and did not differ in carotenoid, tocopherol, and chlorophyll contents (Figure S3), confirming that neither seedling growth nor nutritional quality was affected by the spaceflight.

### 3.2. Effect of Controlled Artificial Ageing on Earth and Space Seed Germination

**Figure 2:**
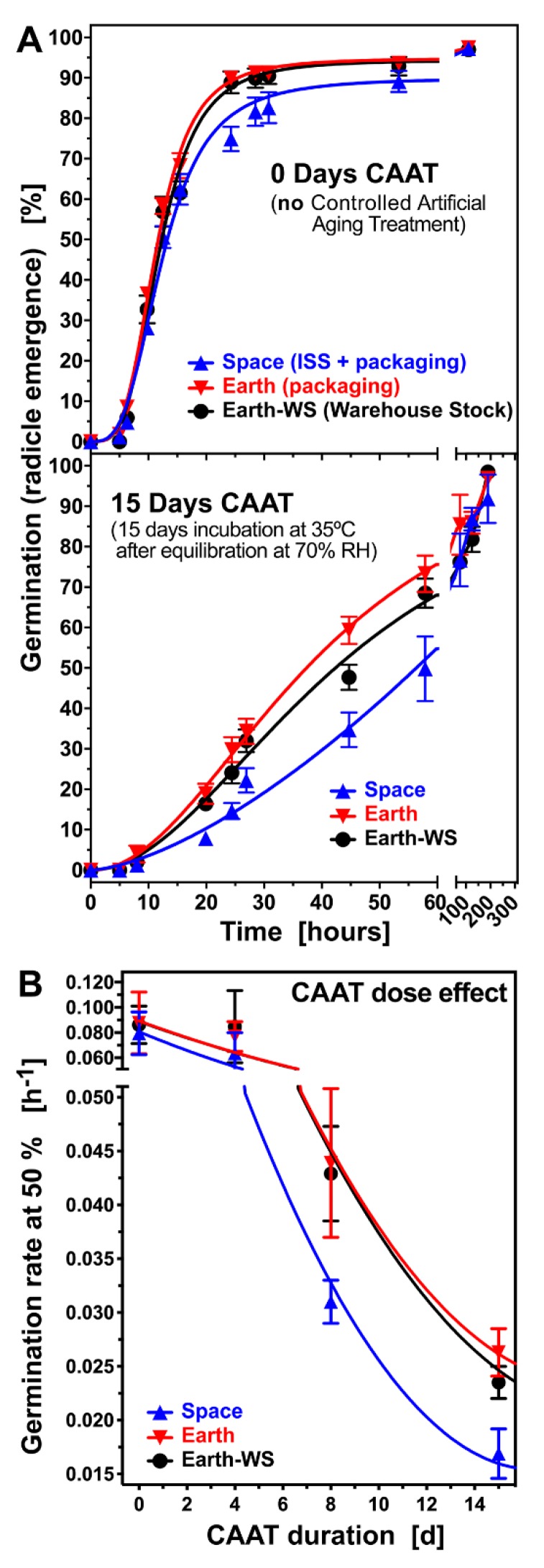
Effect of controlled artificial ageing treatment (CAAT) on Earth-WS and Earth compared to Space rocket seeds. (**A**) Time course of germination of untreated (no CAAT applied) seeds and time course of germination in seeds exposed to 15 days of CAAT. (**B**) Effects of 4, 8, and 15 days CAAT on the germination rates of seeds. CAAT was at 70% RH and 35 °C; subsequent germination scoring was at 20 °C. Mean of three replicates of 20 seeds per plate. Error bars indicate ± standard error.

Due to the delay in germination observed, it was hypothesized that the Space seeds may have experienced an environment that slightly accelerated ageing-related processes. The effect of the controlled artificial ageing treatment (CAAT) on germination was therefore analyzed on all three seed batches: Earth-WS, Earth, and Space seeds (Figure 2). The CAAT involved incubating seeds equilibrated at 70% RH for a defined number of days at 35 °C. Subsequently, they were dried back over silica beads, and their responses were compared in germination assays at 20 °C. Again, when imbibed at 20 °C without CAAT, the Space seeds germinated slightly slower than the Earth-WS and Earth seed batches (Figure 2A *top panel*), on average reaching 50% germination 1.1 h later. When artificial ageing was applied, 15 days of CAAT increased the time to reach 50% germination (t_50%_) of Earth-WS and Earth seed batches by 34.4 and 26.6 h, respectively (Figure 2A *bottom panel*). However, the t_50%_ of the Space seed batch was most affected by the 15 day CAAT, being increased by 46.4 h (Figure 2A *bottom panel*). The Space seed batch was already significantly more susceptible to CAAT at eight days of CAAT (Figure 2B). The dose effect reveals that increasing CAAT duration decreased the germination rates GR_50%_, with Space seeds being more sensitive compared to Earth and Earth-WS seeds. For Space seeds, the t_50%_ was 1.2, 2.7, 9.5, and 21 h longer than for the Earth seeds following 0, 4, 8, and 15 days of CAAT treatment, respectively (Figure 2B). Thus, the difference in germination kinetics between earthbound seeds and space-flown seeds is augmented following the CAAT. The higher ageing sensitivity of the Space seeds was evident in a reduced speed of germination.

Seed ageing during storage reduces seed quality initially by reducing the vigor (speed and uniformity of germination) and only subsequently by reducing the viability of a seed population [58]. Consistent with a loss in seed quality due to enhanced ageing, the Space seeds were reduced in their germination speed but not affected in their viability as evident from the retained maximum germination percentage of 95–100% (Figure 2). To further investigate the seed ageing, we employed an electrical conductivity (EC) vigor assay to compare leakage of solutes from seeds during imbibition [38]. Figure S4 demonstrates a significantly higher EC for Space seeds as compared to Earth seeds. At 24 h soaking time, the Space seed EC was ~1.3-fold higher. This finding further supports our conclusion that the Rocket Science space travel with six months on board the ISS has caused a reduction in seed vigor, with reduced speed of germination and increased ageing sensitivity, but without affecting seed viability. 

### 3.3. Overall Effect of Spaceflight and Controlled Accelerated Ageing on the Seed Transcriptomes

In light of the effects of spaceflight on seed germination kinetics, the transcriptomes of Space and ground control (Earth and Earth-WS) seed batches were investigated to see if transcript abundance changes may suggest mechanisms by which spaceflight caused the reduced germination vigor and increased the ageing sensitivity. Further, because the difference between germination of the ground control and Space seed batches was largest following 15 days CAAT, their transcriptomes upon ageing were comparatively investigated. This revealed potential ageing-related processes involved in the effect of spaceflight and allowed determination of whether the ageing response of space-flown seeds differs to ground control seeds. Thus, the transcriptomes of the three seed batches (Space, Earth, Earth-WS), ‘dry seed’ without or with 15 days CAAT, were comparatively analyzed (six treatments, each with three replicates). 

**Figure 3:**
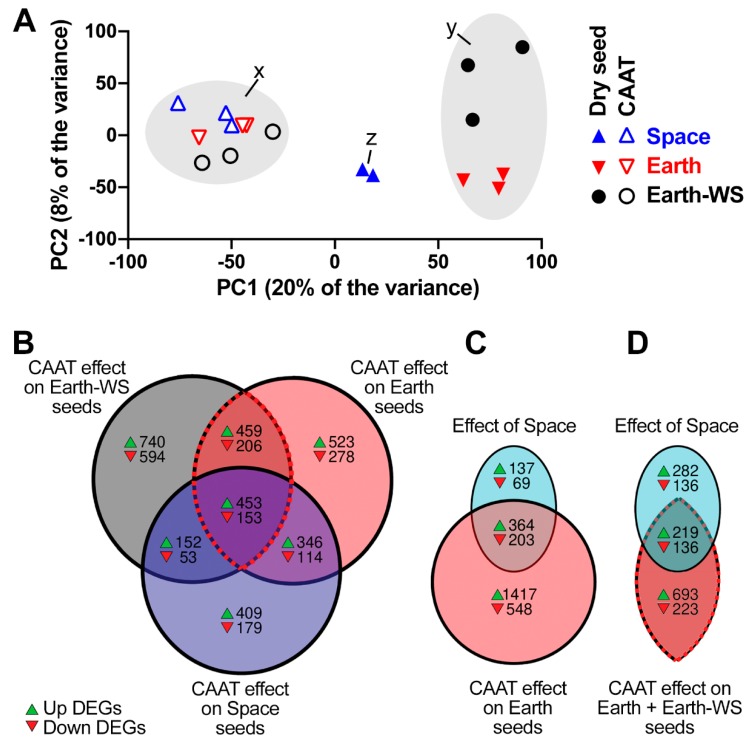
Similarities between the effects of controlled artificial ageing treatment (CAAT) and spaceflight on the rocket seed transcriptomes. (**A**) PCA of gene expression from Earth-WS, Earth, and Space seeds, dry or after 15 days CAAT. ‘x’ indicates aged seed group, ‘z’ indicates space dry seed, and ‘y’ indicates the earthbound dry seed group. (**B**) Venn diagram showing overlap of differentially expressed genes (DEGs) between dry and CAAT-treated seeds across the three seed lots based on comparisons outlined in Table 1. (**C**) Venn diagram showing overlap of DEGs between ageing of Earth seed and DEGs due to spaceflight. (**D**) Venn diagram showing overlap of consistent ageing DEGs (CAAT effect on Earth and Earth-WS seed outlined with red and black dashed line) and DEGs due to spaceflight.

Because the *E. sativa* genome was not available, a de novo assembly was used as a reference for transcript quantification. Following filtering steps, the expression results for 26,234 transcripts were obtained and are accessible via the Rocket Science Gene Expression Viewer (Supplementary Data File); detailed instructions for using the Expression Viewer are described (Supplementary Instructions). One sample, Space—dry seed replicate 2, was removed from the analysis as it was a clear outlier (Figure S5). The PCA of transcript expression (Figure 3A) indicated that ageing affected the transcriptomes. The first principle component, associated with the CAAT treatment, accounted for 20% of the variance in transcript expression. The second principle component was not associated with a specific treatment, although the Earth-WS and Earth seeds were separated along this axis, perhaps indicating that this 8% of the variance might be in part associated with the packing and storage element experienced by the Earth and Space seeds (Figure 3A,y). The three seed batches appeared to be transcriptomically more similar following 15 days CAAT (Figure 3A,x). Interestingly, the Space seed samples (Figure 3A,z) were located between the Earth dry (Figure 3A,y) and the aged seed groups (Figure 3A,x). Thus, we speculated that the spaceflight may have a partial ageing effect on the dry seed transcriptome.

To investigate the genes which contribute to the transcriptomic differences between the treatments, differentially expressed genes (DEGs) between pairwise comparisons of treatments were identified using NOISeqBIO (Table 1). In general, the CAAT induced the most changes in the transcriptomes (2810, 2532, 1859 DEGs in Earth-WS, Earth, Space seeds, respectively). There was a modest overlap in DEGs due to the CAAT across all three seed batches (Figure 3B), with 453 genes consistently induced and 153 repressed by the ageing treatment. A number of genes were also consistent DEGs in two out of the three CAAT comparisons, meaning 42% of all identified DEGs due to CAAT were consistent in at least two seed batches, and 13% were consistent in all three seed batches. The two ground control seed batches shared 45% of DEGs due to CAAT. Overall this indicated that there were consistent effects of CAAT on the transcriptomes, but also seed batch-specific effects. The effects of packaging and spaceflight on the transcriptomes on the dry seed were overall less pronounced than the effects of CAAT (Table 1). Space and packaging, i.e., Space vs. Earth-WS resulted in the most DEGs in comparisons of dry seed (1313). Packaging of Earth (seed storage at ~22 °C) vs. nonpackaged Earth-WS (seed storage at 14 °C) seeds resulted in 942 DEGs in dry seeds. Because Earth and Earth-WS seeds were both not aged and did not differ in ageing sensitivity, we did not focus on these genes in this publication. Space alone, i.e., Earth vs. Space, resulted in 773 DEGs. The differences amongst the seed lots following CAAT were the smallest, with the largest difference in CAAT seed still maintained between Space and Earth-WS with 388 DEGs (Table 1). The Space and Earth seed batches were relatively very similar following CAAT, with only 44 DEGs between Space CAAT and Earth CAAT treatments. It is also evident in the trends of PCA that the CAAT aged seeds are more similar to each other and that CAAT has a fairly consistent effect (Figure 3A).

Notably, Space seeds exhibited fewer DEGs than the ground control (Earth, Earth-WS) seeds following CAAT treatment (Table 1), and the Space samples lie between the CAAT aged seeds and the dry ground control seeds in the PCA (Figure 3A), again suggesting a potential overlap between the effect of space and ageing on the transcriptome. Indeed, it was found that 73% of DEGs due to spaceflight (Earth dry vs. Space dry) were also amongst the DEGs due to ageing of Earth seed, although conversely only 22% of the DEGs due to CAAT were represented in DEGs due to space (Figure 3C). This was indicative that the effect of spaceflight can be explained in part by ageing effects, but that the seeds may only be partially aged, and also that there are some space-specific effects not explained by ageing. To confirm that the effect of spaceflight was consistent with ageing, it was compared to the consistent effect of CAAT (seen in Figure 3B with the red outline) on both Earth and Earth-WS seed batches (Figure 3D). In this case, seed ageing could explain 46% of space-induced DEGs, and space-induced DEGs represented 24% of seed ageing DEGs. This reinforced the hypothesis that the space-effect on the transcriptome may in part reflect a partial seed ageing effect which may explain the observed seed responses. 

### 3.4. Functional Gene Groups Associated with Transcriptome Changes during Ageing and Spaceflight

**Figure 4:**
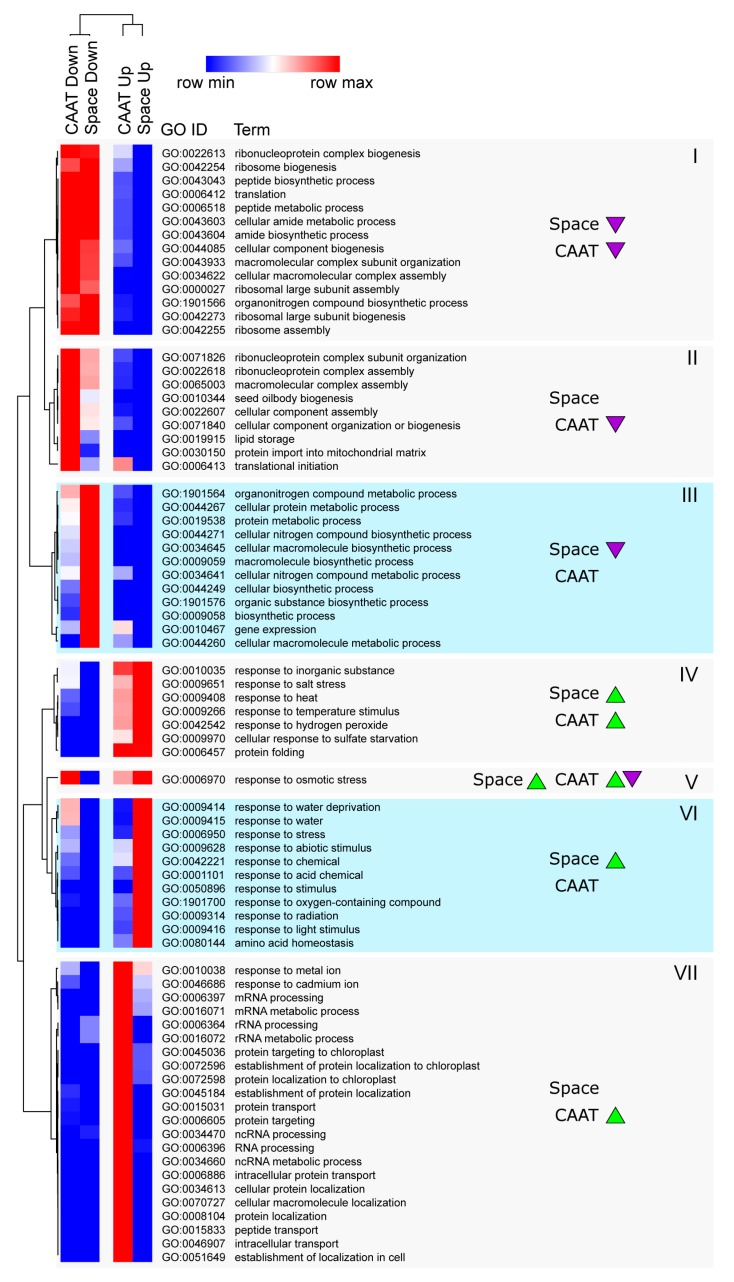
Comparison of gene ontology (GO) term enrichment in space- and CAAT-induced DEGs from rocket seed transcriptomes. GO terms with minimum enrichment scores above 4.8 in at least one DEG list were hierarchically clustered by their enrichment scores in the four DEG lists: Up in Space, Down in Space (Earth dry vs. Space dry), Up in CAAT, and Down in CAAT genes (Earth dry vs. Earth CAAT). Seven clusters (I to VII) are shown and annotated with the direction of expression levels for space and CAAT treatment associated with the GO terms (up or down arrows).

To determine the general functional classes of genes associated with ageing and space-induced transcriptome changes, gene ontology (GO) term enrichment analysis was performed (Figure 4). Similar to the overlap seen with CAAT and spaceflight-induced DEGs, there was also overlap in the functional categories associated with genes up- and downregulated by spaceflight and CAAT. In particular, terms associated with translation are enriched in both genes that are less expressed following ageing (‘Down in CAAT’) and genes that are less expressed following spaceflight (‘Down in Space’), as shown in cluster I (Figure 4, *‘Down’ indicated by arrows*). However, some terms were more strongly associated with a reduction in gene expression following either spaceflight or ageing. For example, terms in cluster II such as ‘seed oilbody biogenesis’ were more associated with a reduction in gene expression following CAAT. On the other hand, the term gene expression and others in cluster III were more associated with a reduction in expression following spaceflight (Figure 4). Conversely, some GO terms were associated with upregulation following both spaceflight and CAAT (clusters IV and V), including terms such as ‘response to heat’ and ‘protein folding’ (Figure 4, *‘Up’ indicated by arrows*). For the GO term ‘response to osmotic stress’ in cluster V the pattern was a bit more complicated, as this GO term was enriched in both genes that were associated with an increase in expression and genes that were associated with a decrease in expression following CAAT. This suggests that a number of genes belonging to this GO term category were induced by CAAT and a number of other genes belonging to this GO term category were repressed by CAAT. Cluster VI represented GO terms that were mostly associated with genes that increased in expression only following spaceflight and included terms such as ‘response to stress’, ‘response to light’, and ‘response to radiation’ (Figure 4). Alternatively, Cluster VII was associated with genes that increased in expression only following CAAT, including the terms ‘mRNA processing’ and ‘protein targeting to chloroplast’. Thus, it was possible to see that there were similarities in the functional annotations of genes that were upregulated or downregulated in spaceflight or following CAAT, suggesting that in part the changes may be as a result of the same mechanism. However, some functional annotations were associated more specifically with spaceflight or with CAAT, suggesting that different mechanisms may also be involved in the two processes. 

### 3.5. Processes Affected at the Transcript Abundance Level by Ageing and Spaceflight

**Figure 5:**
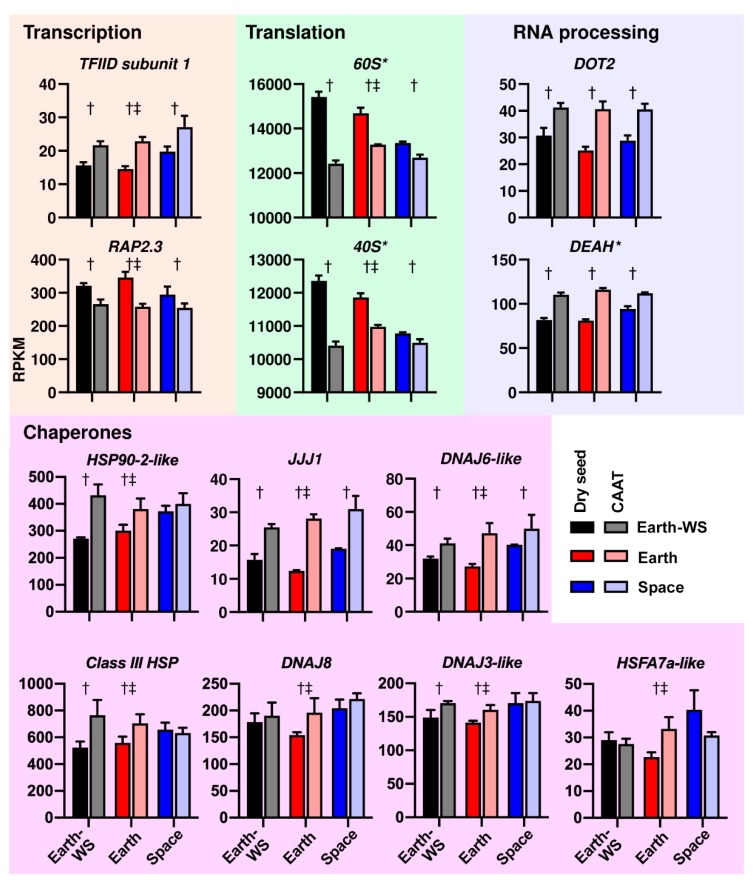
Transcript abundances in RPKM (reads per kilobase of transcript per million mapped reads) of selected genes involved in processes identified in the nontargeted analysis of rocket seed transcriptomes. * Sum RPKM for gene calculated from multiple transcripts. † Significant effect of the controlled artificial ageing treatment (CAAT) and ‡ significant effect of space for at least one transcript belonging to the gene; mean values ± SE are presented. Identity of the transcripts for each gene is provided in Table S2.

**Figure 6:**
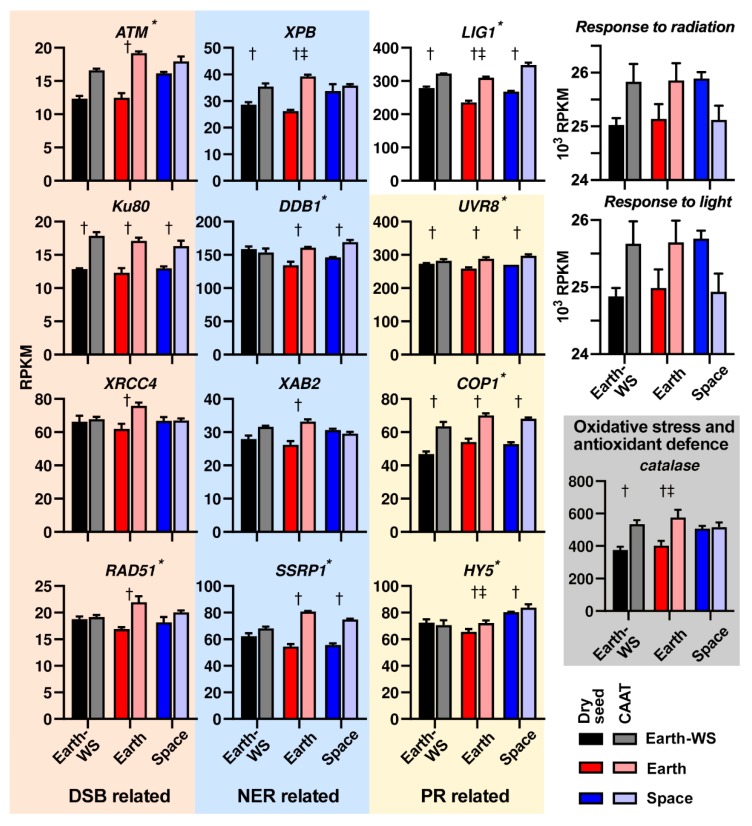
Rocket seed transcript abundances of selected genes involved in DNA repair pathways. The expression levels (in RPKM) of genes involved in double-strand break repair (DSB related), nucleotide excision repair (NER related), and regulation of photoreactivation (PR related) are shown. Additionally, *LIG1* (can be involved in DSB and base excision repair related pathways) expression is shown, as is the sum of transcripts annotated with the GO term ‘response to radiation’. * Sum RPKM for gene calculated from multiple transcripts. † Significant effect of ageing treatment and ‡ significant effect of space for at least one transcript belonging to the gene; mean values ± SE are presented. Identity of the transcripts for each gene is provided in Table S2.

To investigate the processes highlighted by the transcriptome analysis in more detail, we assessed a number of DEGs with described functions belonging to the identified GO terms (Figure 5 and Figure 6). One of the most prominent signals seen in the data was the change in abundance of transcripts encoding translational machinery, particularly those related to the 60S and 40S ribosomal subunits. In agreement with the identification of enrichment of the GO term ‘translation’ for the Down groups in CAAT and Space DEG lists (Figure 4), transcripts encoding proteins of the 60S and 40S ribosomal subunits were significantly less abundant in CAAT-treated seeds and were lower in Space dry seeds compared to the ground control (Earth, Earth-WS) dry seeds (Figure 5). Further, the effect of ageing, for example on the 60S-related transcripts, decreases in Space seeds from 19% and 10% CAAT-induced decreases in Earth-WS and Earth seeds, respectively, to a 5% CAAT-induced decrease in Space seeds (in terms of summed transcripts). The same trend is observed in the 40S-related transcripts. This pattern reflects well the position of the Space dry seed in between Earth dry and CAAT aged seeds in Figure 3. The GO term ‘gene expression’ was identified as being more specifically enriched in the DEGs Down group for Space seeds (Figure 4).

Some of these DEGs for this GO term encode transcription factors, such as *RAP2.3,* which follows a similar trend in decreasing due to CAAT and spaceflight (Figure 5). However, components of the transcription machinery are sometimes expressed in a contrary manner. For example, the transcripts encoding subunits of the general transcription factor TFIID, such as *TFIID subunit 1*, were induced by CAAT and spaceflight following the opposite pattern to the ribosomal subunits and *RAP2.3*. Transcripts encoding TFIID subunits 2, 6, 9-like, and 11 were also upregulated (Supplementary Data File). Thus, some elements of gene expression machinery appear to be induced by spaceflight and CAAT, whilst others are repressed. Another element of gene expression, RNA processing, was a GO term identified as being significantly enriched in CAAT-induced DEGs (but not space-induced DEGs), indicating an ageing process that is not as represented in the hypothesized ‘space ageing’ process. Amongst the ‘RNA processing’ GO term genes induced by CAAT were the pre-mRNA splicing proteins DOT2 and four DEAHs (Figure 5) [59]. Their expression was only significantly affected by the CAAT treatment, and not by spaceflight, although there was a slight tendency for elevated *DEAH* expression in dry Space seeds compared to Earth seeds (Figure 5).

Stress response processes were also identified as being important in the CAAT and spaceflight-induced DEGs, including the GO terms ‘response to heat’ and ‘protein folding’. Indeed, a number of heat shock proteins (HSPs) and chaperones [11,60,61] were found to have elevated expression in CAAT aged samples, and in space-flown samples, including *HSP90-2-like, HSP90-1*, *class III HSP*, *JJJ1*, and several *DNAJ*-related transcripts (Figure 5) and other HSPs (Supplementary Data File). For example, *HSP90-1* expression was increased by 53%, 74%, and 38% in CAAT treated Earth-WS, Earth, and Space seeds, respectively. Space seeds had 23% more *HSP90-1* and 31% more *HSP90-2-like* transcripts than Earth seeds (Figure 5). Again, the lesser effect of ageing on expression of *HSP90-1* and *HSP90-2-like* in space-flown seeds reflects the position (in the PCA, Figure 3A) of space-flown dry seeds as being between dry and CAAT seeds. Roles of HSPs in spaceflight responses (Figure 5, [11,60,61]) are further supported by heat shock factor transcript expression patterns, including *HSFA7a-like* in Space seeds (Figure 5) and *HSFA2* [11].

### 3.6. Expression of DNA Repair Related Pathways Following Ageing and Spaceflight

One of the potential mechanisms by which spaceflight and ageing may affect seed physiology is through DNA damage. Transcripts annotated with the GO term ‘response to radiation’ were significantly enriched in genes upregulated in Space seeds particularly, and to a lesser extent by CAAT (Figure 4). This GO term included some genes involved in repairing damage to DNA due to ionizing radiation, such as UV. We thus checked if there was evidence of differentially expressed DNA damage repair mechanisms following space and ageing treatments. DEGs were identified in the double-strand break (DSB) repair, nucleotide excision repair (NER), and photoreactivation-related (PR) DNA repair pathways (Figure 6, Supplementary Data File). In the DSB repair pathway, the DSB signal initiation related *ATM* [25,62,63] was a DEG elevated by CAAT in the Earth samples (Figure 6). Consistent with partial ageing by the spaceflight (see PCA, Figure 3A), *ATM* transcript abundances were higher in dry Space seeds compared to the ground controls (Figure 6). *ATR* transcripts exhibited a similar pattern (Supplementary Data File). *Ku80*, *XRCC4*, and *RAD51* of the DSB repair pathway [25,63,64,65,66] were induced by CAAT, especially in Earth-WS seeds (Figure 6). *DNA ligase 1* (*LIG1*) also functions in DSB repair, as well as in other pathways, including the base excision repair (BER) pathway [25,66,67]. *LIG1* was significantly induced by CAAT by ca. 35% in all seed batches and was also 14% higher in Space as compared to Earth dry seed (Figure 6). 

Expression of components of the NER pathway was also elevated by CAAT and/or spaceflight, including *XPB, DDB1, XAB2,* and *SSRP1* (Figure 6). Whilst photolyases were not significantly affected by CAAT or spaceflight (Supplementary Data File), other components involved in regulating the photoreactivation repair (PR) repair pathway including *UVR8*, *COP1,* and *HY5* [68,69,70] were identified as DEGs (Figure 6). *HY5*, a transcription factor involved in expression of photolyases, was induced by CAAT in Earth and Space seeds; it was also induced by spaceflight in dry Space seeds (Figure 6). Mismatch repair and BER related genes were generally not found to be affected by ageing or spaceflight, with the exception of the aforementioned *LIG1,* which is also involved in BER. On the whole, transcripts encoding other parts of pathways involved in reactive oxygen species (ROS) detoxification (e.g., transcripts encoding glutathione reductase, glutathione *S*-transferase, superoxide dismutase, ascorbate peroxidase, ascorbate reductase, and sulfoxide reductase) [71,72] were not generally induced by CAAT or spaceflight (Supplementary Data File). However, catalase transcripts known to be involved in the recovery from seed ageing [73] were induced by CAAT and in dry Space seeds (Figure 6). Among the DEGs with *Up in Space* patterns in the GO terms ‘response to radiation’ and ‘response to light’ (Figure 6) were at least five catalases, *HSF7A-like*, several HSP- and DNAJ-related chaperones (including those shown in Figure 5), and *HY5* (Figure 6).

## 4. Discussion

### 4.1. Effects of Spaceflight Environmental Factors on Dry Stored Seeds

In the Rocket Science project, a spaceflight including six months on board the ISS in low Earth orbit (LEO) had a subtle, but significant, effect in delaying germination of rocket seeds, consistent with the observations of over 5000 schools across the UK, which also reported lower seedling survival in space-flown seeds at 17 days and reduced seedling height at 21 days [36]. The major finding in our experiments is that the spaceflight caused partial seed ageing that reduced the seed vigor, as manifested in reduced germination rate (speed), increased solute leakage upon soaking, and increased sensitivity to controlled artificial ageing treatment (CAAT), but the spaceflight did not affect seed viability (maximal germination percentage) of the seed population. Our transcriptome analysis of dry seeds and seeds subjected to CAAT revealed footprints of the observed partial seed ageing caused by the spaceflight in that defined pathways and marker genes were affected. Space environmental factors potentially affecting seeds and seedlings include microgravity; different types radiation, such as galactic cosmic rays, solar energetic particles, and short-wavelength UV; atmospheres with distinct gas composition including the lack of oxygen; low pressure and low humidity in extreme vacuum causing ultradrying; extreme temperature fluctuations; and mechanical vibration during spaceflight [1,5,11,74,75]. While our experimental setup did not explicitly allow discrimination of the individual environmental factors that caused the observed seed ageing, the fact that we investigated seeds in a dry state at low seed moisture content and that they were not exposed to the outside space environment did allow a critical discussion of the expected importance of each of these space factors.

Rocket species, including *E. sativa*, are commercially important salad crops cultivated worldwide [76]. It is therefore an important plant for nutrition and food (fresh produce) during spaceflights [12,13,15,16,17]. Colla et al. (2007) demonstrated that rocket seed germination and seedling growth on board the ISS was affected in that the maximum germination percentage was reduced from 95% to 80% and that subsequent seedling growth and nutrient content were negatively affected. In contrast to their experiment, which included germinating rocket seeds in microgravity on board the ISS, in our Rocket Science project dry rocket seeds were subjected to the spaceflight and no germination experiments on board the ISS were conducted. Known mechanisms for gravity sensing and signaling are evident just after the completion of seed germination and in growing seedlings [2,4,5]. The dry seed state is not gravity-sensitive, as water- and hormone-controlled processes are needed for gravity responses. In agreement with this, we did not observe a reduction of the maximum germination percentage of our Space seed batch and also did not observe abnormal seedlings, confirming the retained seed viability. We demonstrate that when rocket germinated in earthbound conditions, the Space seeds developed into normal and nutritious seedlings rich in carotenoids, chlorophyll, and antioxidants (Figure S3). The microgravity experienced by the dry rocket seeds therefore does not seem to be a major factor for the observed seed ageing and seed vigor reduction.

The space factors of gas, pressure, and humidity are also unlikely to play a major role in our Rocket Science experiment because the seeds were stored in a low-hydrated state in hermetically sealed packs (at 25% RH) inside the ISS (Figure 1A). During the entire spaceflight, the seeds were transported at 18–25 °C (Figure S1), but because no temperature datalogger was used we cannot rule out occasional fluctuations beyond this temperature range. What is however clear is that the seeds did not experience temperatures high enough to reduce their viability. They were retained in a dry state at low humidity and low oxygen conditions at ~22 °C ambient temperature on the ISS (and 18–25 °C during the other parts of the space journey), which provides good storage conditions for maintaining viability of this species for several years [18,19,37]. The warehouse storage (Figure S1) of our Earth (~22 °C) and Earth-WS (14 °C) seeds was also in hermetically sealed packs at low humidity and low oxygen, and neither of the two temperatures caused seed ageing during dry storage. Even if the seeds would have experienced temporarily hotter temperatures (e.g., ~30 °C), this is not expected to severely affect rocket seed quality as *Eruca* species are adapted to the Mediterranean region [76], have their germination optimum at ~30 °C (Figure 1D), and remain viable even when stored in the dry state at 45 °C for several months [37]. Mechanical vibration can damage [77] or improve [78] seed germination. These works demonstrated that vibration in the imbibed state increased both the speed and maximum percentage of Arabidopsis germination and that, in the air-dry state, the mechanical damage which reduced the germinability depended on the seed moisture content. The observed increased equilibrium seed moisture content of the rocket seeds from the Space batch as compared to the Earth and Earth-WS batches (Figure S2) may be due to spaceflight-induced alterations of the biomechanical seed properties. Mechanical vibration, acceleration, and very high sound levels from the take-off, landing, and spaceflight may indeed act synergistically together with space radiation to cause the observed ageing of the Space seeds.

Shielding provided by the ISS during the six months of rocket seed storage in LEO excluded the exposure to short-wavelength UV radiation known for its negative effects on seed lot viability [1,30,31]. Excluding space UV leaves exposure to ionizing radiation from galactic cosmic rays, trapped protons, and solar energetic particles which together provide an absorbed dose of ~500 µGy/day in LEO [30,74,75,79,80]. Shielding reduces this to 250–300 µGy/day inside the ISS, which is much higher than the Earth surface (ground control) at 1–3 µGy/day. Precise simulation of the complex space non-UV radiation is not possible, but dose–response experiments with low linear energy transfer ionizing radiation (e.g., X- and γ-rays) demonstrated for the irradiation of dry tomato seeds that absorbed doses of up to ~300 Gy did not appreciably affect the maximal germination percentage [81,82]. However, the height of tomato plants grown from irradiated seeds was reduced for absorbed doses above ~50 Gy, while low absorbed doses of ~10 Gy increased plant growth. For maize seeds above ~100 Gy caused a reduction in the maximal germination percentage and also reduced plant growth [83]. No reduction in the maximum germination percentage was observed when dry Arabidopsis seeds were γ-irradiated with 100–1000 Gy, but DNA damage and seedling defects were observed at 100 Gy and increased in a dose-dependent manner [60]. Mixed results were obtained for seed storage in the long-duration-exposure facilities of satellites in LEO, resulting in reduced, equal, or improved germination and survival compared to seeds stored on Earth [1,5]. The shielded storage of tomato seeds for 69 months was in sealed canisters (0.1 MPa, 20% RH, temperatures fluctuating between −25 and 35 °C) causing ionizing space radiation exposure of ~7.2 Gy [84]. Compared to the Earth-based control (0.1 MPa (normal pressure), 20% RH, 21 °C), the space-flown seeds germinated slightly quicker and had higher seedling emergence and growth, indicating that tomato seeds were resilient to such spaceflight. Similar space radiation exposure of maize seeds did not appreciably affect their maximal germination percentages but increased their sensitivity to other forms of radiation and caused a somatic mutagenesis equivalent to ~6.4 Gy [85]. Tomato seeds stored in on board the space station MIR for six years (~1 Gy) exhibited reduced seedling growth and a >10-fold increase in DNA mutation rate [86]. Exposure of rice seed lots to LEO in containers attached outside the ISS for 13 and 20 months reduced their maximum germination percentage and affected their dry seed transcriptome. It was proposed from this work that damage to long-lived mRNAs stored in dry seeds causes the change in germination properties [33]. For our Rocket Science project, six months of storage on the ISS would equate to ~50 mGy [74,75,80] for the Space seeds which is a low absorbed dose compared to the described examples, but compared to the Earth seeds it is >>100-times more absorbed radiation. Taken together we therefore propose that, perhaps synergistically with mechanical vibration, the exposure of the dry rocket seeds to this low-level ionizing radiation from galactic cosmic rays, trapped protons, and solar energetic particles was the major factor that caused the observed seed ageing and transcriptome changes. Future experiments need to be designed in a way that allows explicit discrimination between the individual spaceflight environmental factors.

### 4.2. Effects of Spaceflight Associated Ageing on the Seed Transcriptome

To determine if ageing and space travel shared any parallels in terms of their effects of germination physiology and the transcriptome, RNAseq analysis of dry and controlled artificial ageing treatment (CAAT) rocket seed samples were performed. Both the physiological assays and transcriptome analysis pointed towards a potential partial ageing effect of the spaceflight. Compared to the CAAT, the effects of spaceflight were relatively subtle. For example, the delay in germination caused by spaceflight was less than the effect of eight days of CAAT but greater than the effect of four days of CAAT. Most intriguing for the global changes was that the space-flown dry seed transcriptomes were positioned between the dry seed ground controls and the CAAT samples in the PCA analysis (Figure 3A). Overlaps in DEGs between CAAT and spaceflight were observed, as was similarity in functional categories of the genes. However, the effect of CAAT was more pronounced, leading to a large number of CAAT-specific DEGs; in addition some spaceflight-specific DEGs were observed. It may be speculated that the degradation of stored mRNAs involved in seed germination in space-flown seeds, as in ageing, may contribute to the delay in germination. A similar hypothesis is proposed for the effect of exposure to space outside the ISS in delaying and reducing rice germination, where degradation of long-lived seed mRNAs was observed [33]. To our knowledge no other dry seed storage work has been published presenting the effects of spaceflight on the dry seed transcriptomes. Although the physiological and mutation effects of dry seed irradiation (X- and γ-ray simulation experiments) were studied in a considerable number of publications [60,81,82,83], none of them provides a global analysis of the effects on the seed transcriptomes.

Transcriptome analyses of dry-stored seeds have been conducted over various storage periods and ambient conditions (humidity, temperature, oxygen) to investigate their relation to after-ripening and dormancy release as well as longevity, ageing, and viability loss. Long-term seed storage experiments (1–30 years, 35–50% RH, 5 °C) revealed that in many cases RNA integrity (RIN value) of dry seeds decreased over storage time before viability loss [24]. Compared to this work, the six months of seed storage in our Rocket Science project is short, and we also did not observe appreciably reduced RIN values in our Space RNA samples. Various mechanisms for transcript abundance changes during dry after-ripening storage, which releases dormancy and enhances germination, have been proposed [57,87,88,89]. In oilseeds, pockets of higher moisture content in non-oil regions could allow transcription even in low-hydrated seeds [57,87]. *Eruca sativa* seeds contain >40% oil, and the 8.0% overall moisture content of the Space seeds could therefore provide such pockets of higher moisture content distinct from Earth seeds (6.3%) during the various stages of the spaceflight and seed packaging process. Selective degradation of transcripts has also been shown to contribute to transcriptome differences in dry seeds owing to the distinct relative stability of transcripts [24,88,89]. In dry seeds, after-ripening and ageing may both be associated with accumulation of damage caused by the formation of reactive oxygen species (ROS), which cause oxidative damage to nucleic acids (including mRNAs), proteins, and lipids [20,21,71,73]. Bazin et al. (2011) proposed that dormancy release during sunflower seed after-ripening is caused by selective mRNA oxidation. They identified 24 stored mRNAs that became highly oxidized, mainly corresponding to genes involved in stress responses and signaling. Low-energy heavy-ion radiation of dry Arabidopsis seeds induced excess ROS in seeds, delaying germination and negatively affecting seedling growth and the activities of antioxidant enzymes [90]. Irradiation of dry rice seeds with ionizing radiation affected the expression of genes related to ROS scavenging and signal transduction in the derived seedlings [91]. We found in our rocket seed transcriptomes that ROS-related transcripts including catalases are upregulated in Earth samples upon CAAT as well as in the dry seed Space samples without CAAT (Figure 5). Taken together it is therefore possible that oxidative stress caused by the low-level absorbed space radiation during the spaceflight caused the ageing-related transcriptome changes.

Similar to our CAAT, which achieved rocket seed ageing within 8–15 days (70% RH, 35 °C), others used elevated RH and temperature to study the transcriptome and proteome changes during their short-duration artificial seed ageing treatments [72,92,93]. Evidence for de novo transcription and oxidative stress responses was revealed by these studies. A number of parallels between the effects of seed ageing in our study with rocket seeds and a previous study on pea seed ageing can be drawn [72]. For example, one of the strongest signals in both studies is the enrichment of translational machinery (particularly 40S and 60S ribosomal subunits) in genes downregulated by ageing. A similar trend is also observed in the proteome and is suggested to delay protein synthesis, and therefore germination, to allow for nucleic acid damage repair to take place [93]. Indeed, consistent with this and a decreased level of 60S and 40S ribosomal subunit related genes in dry Space seeds and in CAAT Earth seeds (Figure 5), we observe a delay in germination and the relative increase in a number of transcripts associated with DNA repair, perhaps indicating that translation of these stored mRNAs is necessary soon after imbibition. We identified a number of transcripts encoding heat shock protein (HSP) and DNAJ-related molecular chaperones, as well as a heat shock factor (HSF), that were more abundant in dry Space seeds and upon CAAT in all samples (Figure 5). DNAJ6-like and JJJ1 are DNAJ protein chaperones that interact with Hsp70, with the latter being involved in proper assembly of the 60S ribosomal subunit [61]. The protective role of the HSP90 chaperone in γ-irradiated Arabidopsis seeds was demonstrated [60]. An increase in the transcript abundances of nine HSPs was evident in the transcriptomes of dry rice seeds exposed to the space environment [33], and an essential role of HSFs in the adaption of cell cultures to spaceflight was identified [11]. An increase in abundance of transcripts encoding molecular chaperones (including HSPs) was also observed in artificially aged pea seeds [72], and a seed-ageing-related HSF was identified in rapeseed [92]. Seed ageing, including in space and in artificial assays, therefore shares transcriptome responses associated with oxidative stress. Our observation in rocket seeds that some transcript abundances were increased, and others were decreased, in response to spaceflight and ageing treatments could therefore be a combination of de novo transcription and selective oxidative degradation. In any case, the changes in relative transcript abundances affect the behavior of the seeds upon imbibition.

Like other organisms, plants employ a range of DNA repair pathways to ensure faithful replication, proper organization, and transcription, and the mode of repair depends on developmental stage and DNA damage type (reviewed in [63,66]). Repair of DNA damage acquired during dehydration, quiescence, and rehydration is critical in determining successful germination and seedling establishment following rehydration of dry quiescent seeds. Figure 6 shows that the sum of transcript abundances annotated with the GO term ‘response to radiation’ was increased in dry rocket Space seeds, and specific examples are highlighted in this figure and the corresponding main text in the results part. Consistent with a role in the Space seed ageing, the ‘response to radiation’ transcript abundances also increase in Earth seed samples upon CAAT (Figure 6). An example for this is the DNA damage checkpoint kinase ATM, a key factor in the double-strand break (DSB) repair pathway, known to control ageing-induced DNA repair during seed imbibition [62]. In rocket seeds, *ATM* transcript abundance is higher in Space samples and increases in Earth samples upon CAAT (Figure 6). For the nucleotide excision repair (NER) pathway [65,66], a similar pattern is observed for NER components such as *XPB* (Figure 6). *DNA ligase 1 (LIG1)*, involved in DSB and single-strand break repair [25,67] was also enriched by CAAT, although *LIG IV* and *LIG VI*, which are associated with DSB repair in seed longevity and elevation in expression following seed imbibition, were not affected at the transcript level. Some components of the photoactivation repair (PR) pathway are also upregulated in rocket seeds upon CAAT, but except for *HY5* no elevated transcript abundances were evident in dry Space seeds (Figure 6). The UV-B photoreceptor (UVR8) signaling can induce expression of photolyases involved in DNA repair, in part via a HY5-dependent pathway; in the absence of UV-B, COP1 can antagonize this response [68,69,70]. Taken together, the Space rocket seeds were shielded against UV irradiation on board the ISS but experienced low-level ionizing radiation from galactic cosmic rays, trapped protons, and solar energetic particles which may have caused the upregulation of DSB (*ATM*) and NER (*XBP*) DNA pathway transcripts (Figure 6), indicating that DNA is a target of the partial seed ageing.

## 5. Conclusions and Future Directions

Maintaining crop seed quality (vigor and viability) during long-term warehouse storage is a major challenge for the global seed industry and is achieved by reducing humidity, temperature, and oxygen to slow down the rate of seed ageing in the dry state. The resilience of dry seeds was revealed in exposure experiments to the harsh space environment in which their viability was maintained for a considerable time if shielded against the short-wave UV irradiation. Our Rocket Science project results suggest that for maintaining the vigor component of the crop seed quality during long-term spaceflights, additional protection is required. We hypothesize that the observed rocket seed ageing during the space travel, which included six months of dry storage on board the ISS, is caused mainly by exposure to low-level ionizing radiation from galactic cosmic rays, trapped protons, and solar energetic particles, perhaps in combination with mechanical vibration. The absorbed radiation dose on board the ISS would equate to ~50 mGy, >>100-times more than on the Earth surface [74,75,80], and affected the seed transcriptome, germination physiology, and ageing sensitivity. However, long-distance spaceflights would cause much higher exposures for seeds as well as for humans. Calculated exposure on a mission to Mars for example would likely be at least 5-times greater than that experienced by the rocket seed on the ISS. Our study was also limited in that only one seed batch of one species was sent to space and that we did not use a temperature datalogger. Seed storability and ageing resilience, however, differ considerably between seed lots, cultivars, and species, depending on the crop genotype and seed production environment [19]. Thus, careful consideration of seed material, reducing mechanical vibration, and shielding from space radiation would be prudent when taking dry seeds beyond Earth’s orbit, although this study and many others suggest that the goal of growing crops on other worlds is achievable [2,6,7]. 

## Figures and Tables

**Table 1:** Differentially expressed genes (DEGs) in comparisons of the CAAT treatment and comparing the three *Eruca sativa* seed batches (Space, Earth, Earth-WS) in the dry and aged states.

Group	Comparison	A	B	Down in A vs. B	Up in A vs. B
Dry seed vs. CAAT	CAAT effect on Earth-WS seeds	**Earth-WS CAAT**	**Earth-WS**	1006	1804
	CAAT effect on Earth seeds	**Earth CAAT**	**Earth**	751	1781
	CAAT effect on Space seeds	**Space CAAT**	**Space**	499	1360
Dry seed	Effect of Packaging and Space	**Space**	**Earth-WS**	655	658
	Effect of Space	**Space**	**Earth**	272	501
	Effect of Packaging	**Earth**	**Earth-WS**	603	339
CAAT	Effect of Packaging and Space	**Space CAAT**	**Earth-WS CAAT**	45	343
	Effect of Space	**Space CAAT**	**Earth CAAT**	32	12
	Effect of Packaging	**Earth CAAT**	**Earth-WS CAAT**	49	300

## Data Availability

Single-ended Illumina raw reads from this study were uploaded to the NCBI Sequence Read Archive (SRA) with BioProject number PRJNA606141 (https://www.ncbi.nlm.nih.gov/bioproject/?term=PRJNA606141). The transcripts fasta file is available online through figshare depository doi: 10.17637/rh.11872515.

## Supplementary Materials

The following are available online at https://www.mdpi.com/2075-1729/10/4/49/s1: Figure S1: Flow chart depicting the relationship between the three seed batches ‘Earth-WS’, ‘Earth’ and ‘Space’, as well as their storage conditions. Figure S2: Effect of spaceflight on seed moisture content in *Eruca sativa* (rocket). Figure S3: Effect of spaceflight on metabolite contents in *Eruca sativa* (rocket) seedlings. Figure S4: Effect of spaceflight on electrical conductivity (EC) in *Eruca sativa* (rocket) seeds. Figure S5: Principle Component Analysis (PCA) of all samples. Rocket Science Gene Expression Viewer Instructions Supplementary File: Detailed Instruction on how to use the Viewer. Rocket Science Gene Expression Viewer Supplementary Data File: Rocket Science Gene Expression Viewer as large Excel data file. Table S1: Raw counts data of transcripts with GO terms annotation. Table S2: Identity of transcripts presented in Figure 5 and Figure 6.

## Abbreviations

ATM, Ataxia-Telangiectasia Mutated; ATR, ATM and Rad3-related; CAAT, Controlled Artificial Ageing Treatment; EC, Electrical Conductivity; DEAH, DEAH-box RNA helicase; DEG, Differentially Expressed Gene; DSB, Double-Strand Break; GO, Gene Ontology; HY5, Long Hypocotyl 5; HSF, Heat Shock Factor; HSP, Heat Shock Protein; ISS, International Space Station; LEO, Low Earth Orbit; NER, Nucleotide Excision Repair; PCA, Principal Component Analysis; PR, Photo-Reactivation; RIN, RNA Integrity Number; RPKM, Reads Per Kilobase of transcript per Million mapped reads; UV, Ultraviolet; WS, Warehouse Storage.
