# Supplementary material for: Rocket Science: The Effect of Spaceflight on Germination Physiology, Ageing, and Transcriptome of Eruca sativa Seeds
Source: Life (Basel). 2020 Apr 24;10(4):49. doi: 10.3390/life10040049 (PMC7235897; doi:10.3390/life10040049)
Supplement: Supplementary file 1 [file life-10-00049-s001.zip › life-754952 suppl/Instructions for Excel expression viewer.docx]

**Instructions for Excel expression viewer**

*Purpose*

To visualize expression and significant differential expression of lists of genes of interest or individual genes of interest in the Rocket Science transcriptome dataset.

For lists of genes, the viewer can display Mean, Mean Z-score or Sum of expression for the genes in a list as a bar plot; a list of significant differences and fold-changes for all genes in the list between samples; and a heat map of all genes in the list and RPKM values. For individual genes, the mean RPKMs and DEGs between chosen comparisons are displayed. Preset lists of genes are incorporated for the standard user.

*Viewing a list of genes*

1. Select a gene list from the first drop-down list (e.g., ‘Earth-WS – Down in CAAT’, a list of genes that are significantly lower in CAAT-treated than dry Earth-WS seed). Many lists of genes are provided. Scroll the list see all options available.

2. Select a summary statistic to plot in the graph on the left (e.g., Mean).

3. To view the genes in the selected list and their status as DEGs, heatmap, and expression values, click the link ‘3) View list of genes’. The list of genes and nearest BLAST hits is shown (A). DEG status in the sample comparisons is shown to the right (B). Further to the right (C), the heatmap (based on Z-scores) (D), RPKM values (E), and means and standard deviation (F) are shown.

4. To return to the main page, click ‘Back to Graph and List Select’ (G).

5. If automatic calculations are disabled (recommended), then it is advised to recalculate (in Windows, F9, otherwise one may click ‘calculate’ in the bottom left corner) in Excel after each step. If slow performance is observed due to automatic calculations one can disable this in File > Options > Formulas, choosing ‘Manual’ under ‘Workbook Calculation’. Automatic calculations can be re-enabled by selecting ‘Automatic’.

*Viewing a single gene from the list of genes*

1. Select a gene list from the first drop-down list (e.g., ‘Earth-WS – Down in CAAT’, a list of genes that are significantly lower in CAAT treated than dry Earth-WS seed). Many lists of genes are provided. Scroll the list see all options available.

2. Select ‘Select Individual Gene (below)’ from the second drop-down list.

3. Select the single gene of interest. The mean expression for the treatments will be plotted in the graph on the left. The list may contain many genes. Scroll the list see all options available.

4. The DEG status for the different treatment comparisons will be shown for the selected gene (along with fold changes). This only works when single genes are selected.

5. To view a gene outside the lists, it is possible to follow steps 1 and 2, but then paste the gene ID of interest into box 3.

6. If automatic calculations are disabled (recommended) then it is advised to recalculate (F9) in Excel after each step.

*Notes for advanced users*

By default, sheets are protected, and some are hidden. Unprotecting sheets may be required to copy values if desired. To add custom lists of genes, right-click sheet tabs and select Unhide. Unhide the sheet ‘Lists’. Add a custom list of genes to the end of the sheet ‘Lists’ in the same format as preceding lists. The current viewer only supports viewing lists of 2500 genes without more extensive modification.

| 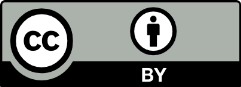 | 1. © 2020 by the author. Licensee MDPI, Basel, Switzerland. This article is an open access 2. article distributed under the terms and conditions of the Creative Commons Attribution   (CC BY) license (http://creativecommons.org/licenses/by/4.0/). |
| --- | --- |
