# Supplementary material for: Rocket Science: The Effect of Spaceflight on Germination Physiology, Ageing, and Transcriptome of Eruca sativa Seeds
Source: Life (Basel). 2020 Apr 24;10(4):49. doi: 10.3390/life10040049 (PMC7235897; doi:10.3390/life10040049)
Supplement: Supplementary file 1 [file life-10-00049-s001.zip › life-754952 suppl/life-754952-suppl-figures.docx]

Supplementary Material for Rocket Science: The Effect of Spaceflight on Germination Physiology, Ageing, and Transcriptome of *Eruca sativa* Seeds

Jake O. Chandler ^1^, Fabian B. Haas ^2^, Safina Khan ^1^, Laura Bowden ^3^, Michael Ignatz ^1^, Eugenia M. A. Enfissi ^1^, Frances Gawthrop ^4^, Alistair Griffiths ^5^, Paul D. Fraser ^1^, Stefan A. Rensing ^2^ and Gerhard Leubner-Metzger ^1,6,^*

^1^ Department of Biological Sciences, Royal Holloway University of London, Egham, TW20 0EX, United Kingdom; jake.chandler@rhul.ac.uk (J.O.C.); k_safina@hotmail.com (S.K.); ignatz.michael@gmail.com (M.I.); [Genny.Enfissi@rhul.ac.uk](mailto:Genny.Enfissi@rhul.ac.uk) (E.M.A.E.); P.Fraser@rhul.ac.uk (P.D.F.);

^2^ Plant Cell Biology, Faculty of Biology, University of Marburg, 35043 Marburg, Germany; fabian.haas@biologie.uni-marburg.de (F.B.H.); stefan.rensing@biologie.uni-marburg.de (S.A.R.)

^3^ Official Seed Testing Station for Scotland, SASA, Edinburgh, EH12 9FJ, United Kingdom; Laura.Bowden@sasa.gov.scot

^4^ Tozer Seeds Ltd, Cobham, Surrey, KT11 3EH, United Kingdom; Frances.Gawthrop@tozerseeds.com

^5^ Science Department, Royal Horticultural Society, Woking, Surrey, GU23 6QB, United Kingdom; alistairgriffiths@rhs.org.uk

6 Laboratory of Growth Regulators, Centre of the Region Haná for Biotechnological and Agricultural Research, Institute of Experimental Botany, Academy of Sciences of the Czech Republic, Palaćky University, 78371 Olomouc, Czech Republic

***** Correspondence: Gerhard.Leubner@rhul.ac.uk;

**Figure S1.** Flowchart depicting the details of the spaceflight and controls of the *Eruca sativa* (rocket) seed storage conditions (temperature, relative humidity (RH)).

**Figure S2.** Effect of spaceflight on seed moisture content in *Eruca sativa* (rocket). Comparative seed moisture contents of. Mean values ± SEM of three replicates for Space, Earth and Earth-WS batches; * indicates statistical difference with *p* < 0.05.

**Figure S3.** Effect of spaceflight on metabolite contents in *Eruca sativa* (rocket).seedlings. Metabolites were quantified in 35-day old seedlings grown at 22 °C. Mean values ± SD of 3 replicates of 14 pooled samples each comprising of 12 seedlings. Student's *t*-test revealed no statistical difference between Space and Earth seedlings for any metabolite.

**Figure S4.** Effect of spaceflight on electrical conductivity (EC) in *Eruca sativa.*(rocket) seeds. EC was measured after soaking seeds for 4, 8, 12 and 24 hours at 20 °C. Mean values ± SEM of four replicates of 100 seeds soaked in 50 mL water.


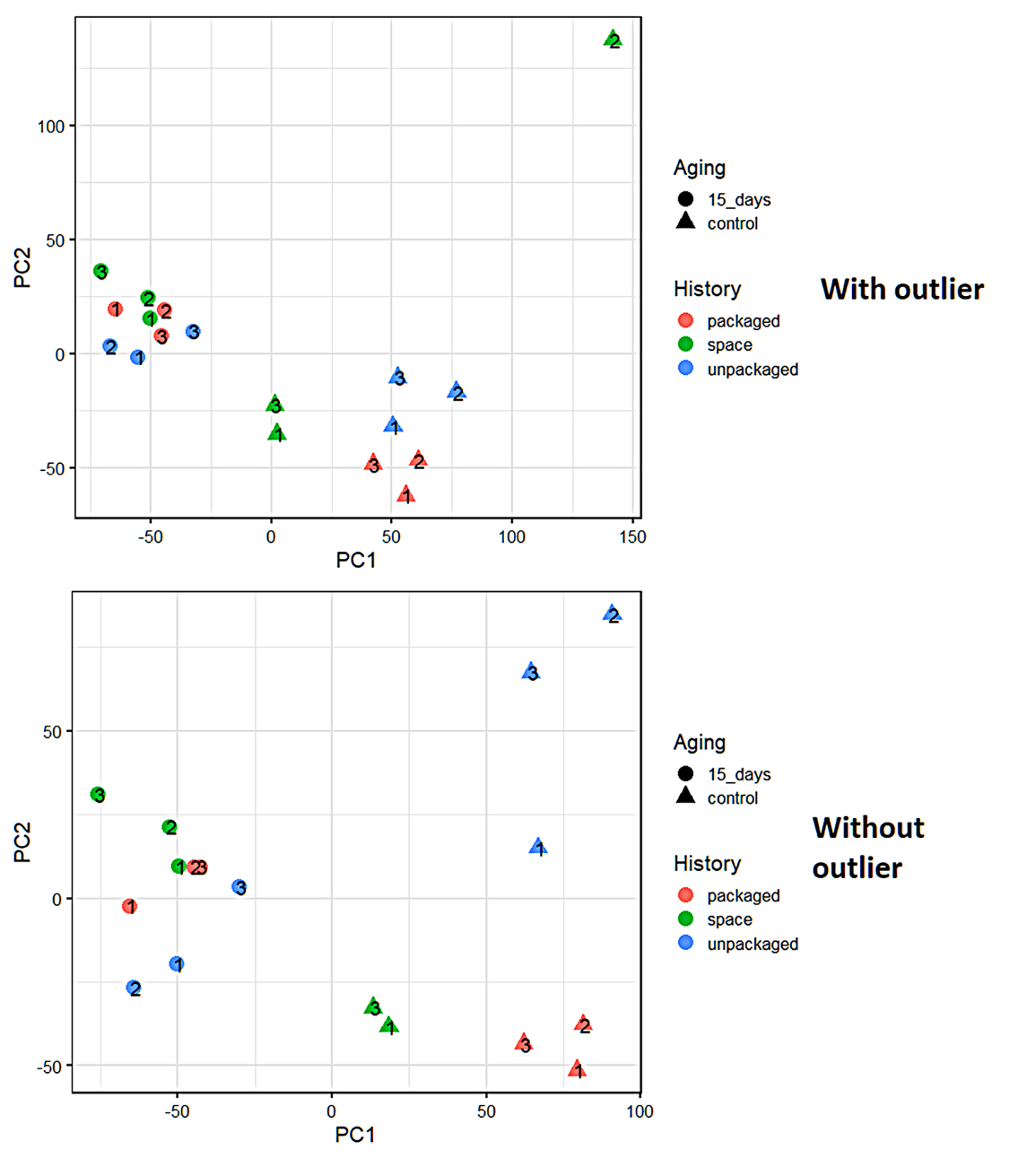


**Figure S5.** Principle Component Analysis (PCA) of all samples identified Space dry replicate 2 as outlier.

| 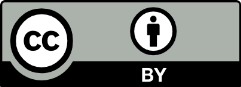 | 1. © 2020 by the author. Licensee MDPI, Basel, Switzerland. This article is an open access 2. article distributed under the terms and conditions of the Creative Commons Attribution   (CC BY) license (http://creativecommons.org/licenses/by/4.0/). |
| --- | --- |
